# A dance movement therapy group for depressed adult patients in a psychiatric outpatient clinic: effects of the treatment

**DOI:** 10.3389/fpsyg.2015.00980

**Published:** 2015-07-10

**Authors:** Päivi M. Pylvänäinen, Joona S. Muotka, Raimo Lappalainen

**Affiliations:** ^1^Tampere Psychiatric Unit, Tampere City Mental Health ServicesTampere, Finland; ^2^Department of Psychology, University of JyväskyläJyväskylä, Finland

**Keywords:** dance movement therapy, depression, antidepressants, treatment outcome, group therapy, psychiatric outpatient clinic

## Abstract

We were interested in investigating the effects of dance movement therapy (DMT) in a psychiatric outpatient clinic with patients diagnosed with depression. DMT aims to engage the patients in physical and verbal exploration of their experiences generated in movement based interaction. The assumption was that DMT, which includes both physical engagement as well as emotional and social exploration, would alleviate the mood and psychiatric symptoms. All adult patients (*n* = 33) included in the study received treatment as usual (TAU). Twenty-one patients participated in a 12-session DMT group intervention, and the remaining 12 patients chose to take TAU only. The majority of the patients suffered from moderate or severe depression, recurrent and/or chronic type. The effects of the interventions were investigated after the intervention, and at 3-month follow-up. Compared to the TAU, adding DMT seemed to improve the effect of the treatment. The effect of the DMT was observable whether the patient was taking antidepressant medication or not. At follow-up, between group effect sizes (ES) were medium in favor for the DMT group (*d* = 0.60–0.79). In the DMT group, the within ES at the 3 months follow-up varied from 0.62 to 0.82 as compared to TAU 0.15–0.37. The results indicated that DMT is beneficial in the treatment of depressed patients.

## Introduction

The global burden of disease studies show unipolar depression as the leading cause of years lived with disability (YLD) in adult population throughout the world (WHO, 2013^1^). In Finland in 2013, mental health problems were the reason for 40% of work disability retirement, and in this group, depression was the most common problem. Further, mental health problems have been the main reason for the early retirement since the year 2000^2^.

In Finland, the Current Care Guidelines^3^ base the treatment of depression on comprehensive diagnostic, clinical, and psychosocial evaluation. The treatment consists of medication and psychotherapy (see Kupfer et al., 2012; Holma, 2013). The recommended brief psychotherapy forms are cognitive, interpersonal, psychodynamic, and problem-solving focused psychotherapy. In practice, medication is often the main intervention to treat depression. It is acknowledged that physical exercise can be beneficial in the treatment of depression, but it cannot replace medication and therapy. Treatment programs in hospital units, day hospitals and outpatient psychiatric clinics may provide some physical activity, and sometimes also dance movement therapy (DMT) is used.

The positive effects of physical activity in the prevention of depression (Brown et al., 2005; Teychenne et al., 2010; Luoto et al., 2013) and in coping with depression (Harris et al., 2005; Rimer et al., 2012) are frequently noted. The Cochrane review on the impact of exercise as a treatment of depression by Rimer et al. (2012) included 39 studies, totaling 2326 subjects. The review indicated that exercise was equally effective as antidepressants or psychological therapies in reducing the symptoms of depression.

Health care providers and sports researchers provide information on amounts of physical exercise that would be the minimum needed to gain the health effects for preventing illnesses, to support the level of functioning in the old age and to foster good mood and happiness^4^. However, a physically active lifestyle is challenged, because the way of living, the methods of transportation and many occupational and leisurely activities are becoming increasingly sedentary. In Finland, collectively, the population is getting less physical activity (Husu et al., 2011) and thus the connectedness to one's embodiment is weakening. Lack of movement and physicality is not only a problem of physical fitness, but also seems to have repercussions on the experiential level, i.e., on the level of body image (Pylvänäinen, 2003, 2012; Koch et al., 2013), which affects social interaction, self-awareness, cognition, and coping. Interestingly, while physical activity in the population has decreased, there are statistical records from the years 1990–2010 documenting a steady increase in the consumption of antidepressants in the Finnish population (Finnish Medicines Agency and Social Insurance Institution, 2012). Patients with depression often suffer from ailments, pain-problems, fatigue, and dissatisfaction with one's own body. When depressed, it is a challenge to overcome the experiential and emotional barriers and reach the benefits of physical exercise and activity. A treatment intervention such as DMT, which includes both physical engagement as well as emotional and social exploration, starting on the level where the patient is, would be feasible to increase self-awareness and emotional and social flexibility among depressed patients (Kiepe et al., 2012; Kolter et al., 2012).

DMT is a form of therapy, which integrates the physical, emotional, cognitive, and social aspects into treatment (Stanton-Jones, 1992; Meekums, 2002; Bloom, 2006; Payne, 2006a; Chaiklin and Wengrower, 2009). DMT aims to engage the patients in physical and verbal exploration of their experiences generated in movement based interaction. DMT can be carried out as individual treatment or in groups. It can be applied to various populations ranging from children to the elderly, and from people with severe psychiatric problems to high-functioning people, who may be interested in strengthening their resources and self-development.

One focus in DMT is engaging with movement: becoming concretely involved in movement activity in the here and now. The other locus of activity is to be attentive to the movement experiences and to develop the skills to be conscious and reflective of them and to communicate about them in words. The relevant interactional elements in DMT are the engagement of moving body, the development of body awareness and mindfulness, and the verbal reflection of the movement experiences, which focuses on the qualities of the experience (Meekums, 2002; Capello, 2009; Koch and Fischman, 2011; Nolan, 2014). It is assumed, that this enables the patient to connect with the emotional core of his/her experience.

The early meta-analysis of the effects of DMT by Ritter and Low (1996) included five studies on people with depression. Two of these studies included psychiatric patients. Revisiting this meta-analysis, Koch et al. (2007) summarize DMT outcome research on depression in a conclusion that the effect sizes in the treatment of depression have ranged from moderate to strong. A Cochrane review of the effects of DMT on depression by Meekums et al. (2015) examined the effects of DMT for depression compared to no treatment or to standard care, to psychological interventions, drug treatment, or other physical interventions. Only three studies met the Cochrane review inclusion criteria, totaling 99 adult subjects and 40 teenage subjects. When the authors compared group DMT to standard treatment in adults with depression, DMT reduced symptoms of depression at follow-up measure, as indicated by clinical observation using the HAM-D. Due to the poor methodological quality of the studies and small sample size, the findings of the effectiveness of DMT could not be considered conclusive. A recent meta-analysis of the effects of DMT and dance on health-related psychological outcomes included the evidence of 23 primary studies (Koch et al., 2014). The meta-analysis showed moderate effects for quality of life and for depression and anxiety.

In the treatment of psychiatric patients the impact of DMT has been positive on body image, the perception of the body and self, affect, motility and well-being, perception of relationships, and biography (Koch et al., 2007, 2014). Goodill's (2005) review of the DMT outcome research in clinical populations concludes that the treatment brings favorable changes in the following dependent variables: vitality, mood, anxiety, mastery, coping-skills, and body image.

Punkanen et al. (2014) conducted a pilot study where DMT group was used in the treatment of depressed patients. Twenty-one depressed adult participants were recruited to participate in 20 sessions of group DMT, twice weekly. The psychometric questionnaires were taken before and after the intervention. The mean score of the primary outcome measure, the BDI, decreased significantly from the pre- (*M* = 21.67, *SD* = 5.26) to post-measurement (*M* = 10.50, *SD* = 5.50), showing that the short-term, group DMT intervention had a positive effect on patients with depression.

As depression is so widespread in the population, it is important to develop its treatment, and if possible, to augment the choices of effective treatments. Research on a current clinical practice in a natural setting is relevant for improving the treatment of depression. Thus, for the development of outpatient psychiatric care, we were interested in investigating the effect of DMT in an outpatient psychiatric clinic. This study plans to add to the knowledge of the effects of DMT in the treatment of psychiatric outpatients diagnosed with depression. The main research question concerned, whether DMT-group intervention produces alleviation in the symptoms of depression. We compared DMT + treatment as usual (TAU) with TAU. Thus, we were interested in whether adding DMT to TAU has benefits as compared with TAU alone. This information may provide legitimation for the choices made on the use of DMT in psychiatric outpatient care.

## Methods

### Recruitment procedure

The research plan was approved by the City of Tampere Research Board, which also is a regional board for ethical research practices. All participants in the study were recruited from a psychiatric outpatient clinic, which is a part of specialized public health care. The patients enter the clinic on a referral from a physician. The patients' treatment is carried out by a multi-professional team, which includes a psychiatrist, a psychiatric nurse, a psychologist, and a social worker. The clinic offers pharmacological treatment, individual counseling, and a selection of group interventions. There are various psycho-educational groups focusing on coping with psychiatric disorder and its symptoms. The DMT group (8–12 sessions) has been one option in the available treatment since 2007. The clinic does not provide physical exercise groups as a treatment option.

Announcements of the study were posted in the lobbies of the clinic. The staff received e-mails about the study, inviting them to tell to patients with depression about the opportunity to participate. The patient information described the study aiming at exploring the treatment of depression and its outcome by comparing TAU and the DMT group intervention.

The inclusion criteria were: depression diagnosis and depression as primary symptom. The exclusion criteria were psychosis, suicide attempts or clear suicide plans, diagnosis of severe personality disorder, diagnosis of current alcohol or substance abuse problem, or debilitating somatic symptoms. Patients entered the study voluntarily and could choose between participating in the DMT group or in the TAU group, where they received the other treatment options the clinic provides. At the clinic, the common practice is that the patient can choose, which of the recommended groups to join. Group participation is never imposed on the patient. Patients participating in the study received information about it, their contribution and freedom to withdraw from the study at any time without consequences for their access to treatment. All the participants in the study were recruited between August 2011 and September 2012 and provided written consent to participate in the study.

Patients joining the TAU group signed the consent, which was then sent to the researcher. The TAU group participants were mailed the set of assessment measures at the start of the research period, after 3 months (12 weeks) and after 6 months since the first measurement point. The replies could be sent in stamped, addressed envelope.

Patients interested in joining the DMT group came to a recruitment interview according to the normal practice. At the end of the interview the patient could decide whether to agree to participate in the research and sign the consent form. After the interview, the set of self-evaluation measures was sent to the patient via mail and s/he mailed them back in a stamped addressed envelope. This procedure aimed at distancing the research aspect of the group and the therapy process. Similarly to the TAU group, the measurements were completed at the start of the intervention period (pre), after the 3-months (12-weeks) DMT intervention (post) and after 3 months (follow-up).

During the data collection period, a total of 25 patients were recruited for the DMT groups. Sequentially, they formed four groups. The therapist/researcher worked with one group at a time. Four patients were excluded from the sample on the basis of the inclusion criteria. Thus, 21 patients could be included in the study, and 19 completed all measures. Two patients did not respond to the self-evaluation measures after the treatment or at the follow-up measurement, but they were included in the statistical analysis. In the DMT group, 84% of the participants stayed in the study and in analyses.

The TAU groups were collected at the same time as the DMT groups. A total of 18 patients joined and provided written consent. Twelve patients answered the pre-measurement self-evaluations and were included in the study. However, only eight patients completed the self-evaluations at all three measurement points. In the TAU group, 67% of the initial participants who completed the first evaluation stayed in the study. Supplementary Figure A summarizes flow of the data collection.

Selecting the sample and assigning the groups this way creates a quasi-experimental research design, as there is no randomization. This limits the validity of the results, but this design was chosen in order to remain close to the everyday practices of the clinic. Also, it was assumed that self-selection to the groups would minimize the drop-out rate in the DMT group.

### Participants

The background information presented on the participants is based on the patient records (see Table 1). About 60% (57.5%) of the participants had two or more psychiatric diagnoses. On the basis of the patient records, in the TAU group the most common diagnoses were F32.1—moderate depressive episode (42%) and F32.2—depression severe/major without psychotic symptoms (25%). In the DMT group the most common diagnosis was F32—major depressive disorder, single episode (29%) and the total percentage of patients with F32-range diagnoses was 43%. In the DMT group, 19% of the participants had an F33 diagnosis—recurrent depressive episode. During the treatment period at the clinic, the medical examination indicated the severity of depression to be moderate or severe in the majority of patients in both groups.

**Table 1:** **Participant data at the pre-measurement—depression characteristics**.

**Descriptives of subjects at pre-measurement**		**DMT group**	**TAU group**	**Total**
*N*		21	12	33
Gender	Male	5(23.8%)	4(33.3%)	9(27.7%)
	Female	16(76.2%)	8(66.7%)	24(72.7%)
Age M (SD)		42(12.7)	38(10.4)	41(11.9)
		Min 20	Min 22	Min 20
		Max 59	Max 55	Max 59
Number of diagnosis	1	7(33.3%)	7(58.3%)	14(42.4%)
	2	10(47.6%)	4(33.3%)	14(42.4%)
	3<	4(19.1%)	1(8.3%)	5(15.1%)
Severity of depression (psychiatrist's recorded assessment)	Mild	5(23.8%)	1(8.3%)	6(18.2%)
	Moderate	9(42.9%)	7(58.3%)	16(48.5%)
	Severe	5(23.8%)	4(33.3%)	9(27.3%)
	Not assessed	2(9.5%)	0(0.0%)	2(6.1%)
Years since first episode of depression	1	1(4.8%)	4(33.3%)	5(15.2%)
	2–3	4(19.1%)	1(8.3%)	5(15.2%)
	4–8	11(52.4%)	3(25.0%)	14(42.5%)
	9–25	5(23.8%) *M* = 7.9 years	4(33.3%) *M* = 6.4 years	9(27.1%) *M* = 7.4 years
Significant relational stress in history or currently	Yes	20(95.2%)	12(100%)	32(97.0%)
	No	1(4.8%)	0(0%)	1(3.0%)

In the whole group, there were five patients, whose primary diagnosis was of anxiety or eating disorder or in the personality disorder range. This reflects the common clinical situation in specialized psychiatric care, that patients' depression is rarely just plain depression. This is also reflected a in the second diagnoses the patients had. Of the whole group 58% had a second diagnosis. Twenty-four percent of these second diagnoses related to soma: pain, heart, lungs, diabetes, hyperkinesis. Fifteen percent of the second diagnoses related to anxiety. In the whole group, 18% of the patients reported a history of alcohol abuse.

The mean duration of time since the first episode of depression was 6.4 years in the TAU group and 7.9 years in the DMT group. The mean length of the current treatment period was 16 months in the TAU group and 21 months in the DMT group. At the pre-measurement, for the majority of the patients, the length of the current treatment period was less than 12 months.

In the TAU group, all the patients were taking antidepressant medication (Table 2). In the DMT group, 57% of the participants were taking antidepressant medication, 43% (nine patients) were not. The difference in the use of medication between the DMT and TAU groups was statistically significant (*x*^2^ = 7.07, *df* = 1, *p* < 0.01). One reason for the referral to the psychiatric unit was a medication resistant depression, where the patient did not benefit from antidepressants. In the DMT group, 38% of the patients were taking some other medication for psychiatric reasons, and in the control group 42%.

**Table 2:** **Participant data at the pre-measurement—treatment features**.

**Descriptives of participants at baseline**		**DMT group**	**TAU group**	**Total**
*N*		21	12	33
Duration of the current treatment period	>6 months	10(47.7%)	5(41.7%)	15(45.5%)
	7–12 months	6(28.5%)	4(33.3%)	10(30.3%)
	13–35 months	3(14.4%)	1(8.3%)	4(12.0%)
	36–96 months	2(9.5%) *M* = 21.1	2(16.6%) *M* = 15.8	4(12.0%) *M* = 14.24
Antidepressant medication	Yes	12(57.1%)	12(100%)	24(72.7%)
	No	9(42.9%)	0(0%)	9(27.3%)
Other psychotropic medication	Yes	8(38.1%)	5(41.7%)	13(39.4%)
	No	13(61.9%)	7(58.3%)	20(60.6%)
Frequency of individual counseling/therapy at pre-measurement	1x/week	1(4.8%)	0(0.0%)	1(3.0%)
	every other week	3(14.3%)	3(25.0%)	6(18.2%)
	every 3–4 weeks	7(33.3%)	5(41.7%)	12(36.4%)
	5 or more weeks interval	9(42.9%)	4(33.3%)	13(39.4%)
	none	1(4.8%)	0(0.0%)	1(3.0%)
Psychoeducational group experience	Yes	4(19.0%)	11(91.7%)	15(45.5%)
	No	17(81.0%)	1(8.3%)	18(54.5%)
Psychotherapy experience	Yes	12(57.1%)	3(25.0%)	15(45.5%)
	No	9(42.9%)	9(75.0%)	18(54.5%)

In the DMT group, 57% of the patients had experience of psychotherapy and in the TAU group 25%. At the pre measurement, in the TAU group 92% of patients had experience of psychoeducational groups and in the DMT group 19%. The difference was statistically significant (*x*^2^ = 16.24, *df* = 1, *p* < 0.01), and was due to the fact that seven patients (64%) in the TAU group were participating in a psychoeducational group for depressed patients during the evaluation of the intervention.

### Intervention procedure

Both the DMT and the TAU group received individual counseling during the study. In the TAU group 33% of the patients had an individual counseling appointment every 5 weeks or less frequently, and 25% had counseling every 1–2 weeks. In the DMT group 67% of the patients had counseling every 5 weeks or less frequently, and 20% every 1–2 weeks.

The DMT intervention was delivered by a psychologist and dance movement therapist trained in the DMT methods of Marian Chase and in authentic movement. The essence of the Chacian approach is engaging in improvised, shared movement, and creating an interactional space through movement (Levy, 1992; Fischman, 2009). The Chacian method is primarily a DMT form of group therapy. Authentic movement, initially developed by Mary Whitehouse and Janet Adler, can be applied as a method in individual or group therapy (Payne, 2006b). The application of authentic movement based practices in DMT in psychiatric outpatient care means emphasizing the non-judgmental empathetic witnessing of movement expression as it appears, the cultivation of conscious awareness of movement, and the allowing of the person to be visible and seen in his/her movement (Adler, 1999; Penfield, 2006). Both the Chacian method and authentic movement promote the integration of intra-actional (within the individual) and interactional (relating with the environment) systems (Capello, 2009).

The DMT group intervention consisted of 12 dance/movement therapy sessions (one session a week for 12 weeks). Each session was 90 min long and included discussion (20–40 min), movement warm-up and process (30–40 min) and a verbal reflection and closure of the movement experience (15–30 min) facilitated by a dance/movement therapist-psychologist. The therapy groups were small with 4–7 participants. The guiding principles for the group facilitation were:

- *supporting the safety in the body* by paying attention to grounding in the movement, body boundaries, respect for personal space, and the mover's position as a modulator of his/her own movement
- *supporting the sense of agency* by emphasizing the choice making in movement, paying attention to the ways one uses one's body in movement and interaction, recognizing the resources the body offers
- *supporting mindfulness skills* by paying attention to the experience of the body sensations, movements, and states, fostering the ability to verbalize these as well as the emotions and imagery relating to the body sensations
- *being attentive to interaction* by paying attention to body responses in the group interaction situations, acknowledging the impact of expectations, and anticipation in the body responses
- *fostering the interaction* by being present and attentive to the patients, conveying seeing and hearing them as they are, respecting the body experience, and encountering via shared movement qualities

As DMT is based on interaction, the group facilitation in practice was an integration of these principles, pre-planned structures and themes, and responses to the needs and themes of the group in the moment. The same therapist working with each group was the constant factor. All sessions included a discussion at the start and after the movement explorations. The discussions were oriented toward expressing embodied experiences and reflecting on them. Discussions also echoed the process and needs of the group. Table 3 presents a model of the 12-sessions group process.

**Table 3:** **A group model based on the integration of the four different DMT group processes**.

	**Theme**	**Process exercises**
1	Introduction, start	Circular motion in joints. Improvisation with name gestures. With picture cards, expressing one's expectations of the DMT group.
2	Familiarizing with the space, moving, and collaboration	Exploring the space/room by moving in it in various ways and acknowledging the others. In a dyad, reflecting each other's movement.
3	Safety and agency, playfulness	Recognizing how one directs attention: outwards, inwards. Sensing body boundaries. Moving eyes open or closed. Exploring the spatial options in movement.
4	Playfulness, agency, finding different options	Exploring spine motility. Imagery and improvisation: If you were an animal, how would the animal move? In a circle, moving by holding hands.
5	Intuition, sensitivity	Activation of the body, starting from the feet. Playing with different movement qualities. Mindfulness skills and breathing: sensing one's walking.
6	Relieving achievement pressure	Sensing hands through different movements. Breathing exercises. Bartenieff Fundamentals^*^ basic exercises. Mindfulness skills: breathing and seeing the other. Polarity: familiar and unfamiliar in movement.
7	Boundaries, distances, directions	Activating hands and breathing, sensing body boundaries, sensing center/core also with strength. Movement improvisation with a focus on near space, middle space, far space. Walking in a dyad and sensing the connection. Drawing a picture of one's experience.
8	Space for motion, boundaries, surfaces—balancing being, and action	Self-nurturing movement and moving on the floor level. Bartenief Fundamentals^^*^^ basic exercises. Getting into vertical slowly and through different postures.
9	Emotion—acceptance and agency in one's life and in relation with environment/others	Movement improvisation from the words selected to express one's present state. Exploring earth, water, air, and fire through movement improvisation—expressing and describing associated feelings.
10	What do I need—attention and focusing in action	In a dyad, hand massage. On a tape line, improvising movement in relation to the line; working with a partner who accompanies the movement in the way one asks for.
11	Accepting needs—nurturing, simplicity, freedom	Moving with breath, gradually engaging the whole body. Simple qigong exercise (breath, clear movement pattern, a sense of opening/stretching, focusing). Requesting from a pair something one needs in movement and/or presence. Homework: to write a poem of one's experiences in this group.
12	Closure—what have I learnt?	Activating the body, grounding, being aware of the body. Simple qigong exercise (same as in the session 11) Poems: sharing them, improvising movement on them. Feedback of the process.

* *See Bartenieff and Lewis, 1980*.

### Outcome measures

The background information assessment included the patient's gender, date of birth, diagnosis, duration of illness, severity of depression, use of medication, and the treatment received by the time of answering the inquiry. The researcher/therapist had also had access to the research subjects' patient records. The self-evaluation measures used in the study and reported in this paper were: BDI-II, HADS, SCL-90, and CORE-OM.

BDI-II (Beck Depression Inventory) and HADS (Hospital Anxiety and Depression Scale) measure mood. BDI-II (Beck et al., 1961, 1996; Dozois et al., 1998) measures depressive symptoms. The score range is 0–63. Higher points indicate more severe depression (0–13 indicates no or very few depressive symptoms, 14–19 indicates mild depression, 20–28 moderate depression and 29–63 severe depression). HADS screens for depression and anxiety symptoms (Norton et al., 2013). HADS is indicating symptoms, when the score is above 8 in anxiety (HADS-A) and depression scales (HADS-D), respectively (Bjelland et al., 2002), or when the total score is ≥9 (Kjærgaard et al., 2014). Both BDI-II and HADS are frequently used in clinical assessment of depression.

The SCL-90 (Symptoms Check List- 90) is a psychiatric self-report inventory consisting of 90 questions. The questions assess a wide range psychiatric symptoms, including depression, anxiety, and somatization (Holi, 2003). Many of the symptoms reflect bodily states and autonomous nervous system arousal. A single number representing the severity of the patient's condition is GSI (global severity index), which is the average score of the 90 questions of the inventory.

CORE-OM (Clinical Outcomes in Routine Evaluation—Outcome Measure) shows the patient's experience of his/her mood and interactions with others and environment. It addresses the patient's global distress and portrays the dimensions of well-being, problems, life functioning, and risk for aggressive/suicidal behavior. Between the general and clinical populations, the clinical cut-off point is 10 points (Connell et al., 2007) or as a total mean score for women 1.29 and for men 1.19 (Evans et al., 2002). CORE-OM is sensitive to change in condition. The CORE-OM all-items score has a correlation of 0.81 with BDI-II and 0.88 with SCL-90-revised version. CORE-OM is applicable to a wide range of populations. It can be used for assessing clinical effectiveness of various models of therapy (Evans et al., 2002).

The self-evaluation measurements were presented to the participants at the start (pre-assessment), after 3 months (post assessment), and 3 months after the end of the intervention (follow-up assessment).

### Statistical analysis

Baseline between-group differences in demographic data and pre-treatment measures were analyzed with independent *t*-tests and chi-square tests, or using Mplus statistics (see below). The effects of interventions were analyzed using hierarchical linear modeling (HLM) in Mplus (version 7) (Muthén and Muthén, 2012). The most important advantage in using HLM with full information maximum likelihood (FIML) estimation method instead using repeated measures ANOVA/MANOVA is that it uses all the available information. Thus all participants who started the study (DMT, *n* = 21, TAU, *n* = 12) were included in the analyses. The missing data in HLM&FIML is supposed to be Missing At Random (MAR). ANOVA/MANOVA approach uses listwise deletion requiring that the missing data have to be Missing Completely At Random (MCAR). This listwise deletion has a greater effect on statistical power than the HLM/FIML method. The HLM uses a full information approach, with standard errors that are robust in the case of a non-normal distribution (MLR estimator in Mplus). The analyses were as follows. First, the group x time interaction was tested with Wald test. Secondly, if the interaction was statistically significant the group differences were tested for the intervention period (pre to post), and follow-up period (post to follow-up) separately.

Effect sizes (ES) were calculated as follows. The *between-groups* ES was calculated after the treatment and at follow-up by dividing the difference between the DMT group mean and the TAU group mean by the pooled standard deviation of the two conditions. The *within-group* ES was calculated for both the post- and follow-up measurements by dividing the mean change from pre-measurement by the combined (pooled) standard deviation (SD) (Feske and Chambless, 1995; Morris and DeShon, 2002). Due to possible differences between groups at pre-measurement, between-group ES differences at post- and at follow-up measurements were corrected by the pre-measurement difference. Thus, corrected between-group ES were reported. A *between-group* effect size of 0.2 was considered small, 0.5 was medium, and 0.8 was large. A *within-group* ES of 0.5 was considered small, 0.8 was medium, and 1.1 was large (Roth and Fonagy, 1996; Öst, 2006).

## Results

### Symptom measurements

At the pre-measurement, the groups were statistically significantly different in their BDI-II -scores (DMT group *m* = 25.00, *sd* = 11.70; TAU group *m* = 32.50, *sd* = 7.60; Estimate = −7.50, *p* = 0.026) and CORE-scores (DMT group *m* = 17.00, *sd* = 6.61; TAU group *m* = 20.65, *sd* = 3.55; Estimate = −3.66, *p* = 0.036). BDI and CORE described the depression symptoms and psychiatric condition to be more severe in the TAU group than in the DMT group at the pre-measurement. Based on the HADS and SCL-90 scores, the groups were not statistically significantly different at the pre-measurement.

Symptoms (SCL-90) decreased more in the DMT group than in the TAU group during the study period. When the intervention and follow-up periods were analyzed separately it was observed that SCL-90 scores changed statistically significantly differently in the DMT and TAU groups during the intervention (Estimate = −0.425, *p* = 0.011) but not during the follow-up (Estimate = 0.031, *p* = 0.086). In the HADS scores, there was a trend for a significantly different change over the three measures. During the intervention the scores changed statistically significantly differently between the DMT and TAU groups (Estimate = −6.295, *p* = 0.024), but not during the follow-up (Estimate = 0.741, *p* = 0.714). In the BDI-II- and CORE-scores there was a greater reduction in the DMT group than in the TAU group, but over time, the groups did not change statistically significantly differently (Table 4).

**Table 4:** **Mean scores and standard deviation for depression (BDI-II), anxiety and depression (HADS), physical and psychological symptoms (SCL-90), and global distress (CORE) at pre, post, and 3-month follow-up**.

**Out-come**	**Pre *M* (SD)**	**Post *M* (SD)**	**Fup 3-mo M (SD)**	**Wald test *df* = 2**	***P*-value**
BDI-II				2.93	0.231
DMT	25.00(11.70)	14.89(13.60)	16.24(13.62)		
TAU	32.50(7.60)	28.97(8.65)	29.66(9.85)		
*d*		−0.67	−0.60		
HADS				5.39	0.068
DMT	20.81(7.99)	13.43(10.24)	14.22(9.85)		
TAU	24.58(4.65)	23.54(6.47)	23.15(7.75)		
*d*		−0.97	−0.79		
SCL-90				8.23	0.013
DMT	1.39(0.76)	0.95(0.74)	0.91(0.67)		
TAU	1.59(0.41)	1.58(0.37)	1.53(0.55)		
*d*		−0.70	−0.67		
CORE				4.14	0.126
DMT	17.00(6.61)	11.95(7.96)	12.31(7.02)		
TAU	20.65(3.55)	20.05(4.55)	19.78(6.04)		
*d*		−0.85	−0.73		

*Between-group effect-sizes (d) are also presented (corrected with pre-measurement difference)*.

To assess the size of the treatment effects, effect sizes were analyzed (see Supplementary Table 1). *Between groups* ES showed large differences (*d* ≥ 0.80) at post measurement in HADS and CORE, and medium size (*d* ≥ 0.50) in BDI-II and SCL-90. At follow-up between groups ES were medium in favor of the DMT group (*d* = 0.60–0.79). The difference in HADS at 3-month follow-up was close to large (*d* = 0.79). In the DMT group, the *with-in group* ESs were medium or close to medium size at post measurement BDI-II (*d* = 0.87), HADS (*d* = 0.92), and CORE (*d* = 0.76), and small in SCL-90 (*d* = 0.57). In the follow-up the within ES were medium for HADS (*d* = 0.83) and close to medium in BDI-II (*d* = 0.75). ESs were small for CORE (*d* = 0.71) and SCL-90 (*d* = 0.62). In the TAU group the within ESs were small (BDI-II, *d* = 0.47) or very small (HADS, *d* = 0.23; SCL-90, *d* = 0.02; CORE, *d* = 0.18) at post measurement. The within ESs were also small in the follow-up (BDI, *d* = 0.37; HADS, *d* = 0.31; SCL-90, *d* = 0.15; CORE, *d* = 0.26). Thus, in the DMT group the within ESs at the 3-month follow-up varied from 0.62 to 0.82 as compared to TAU 0.15–0.37.

### Differences between the groups on the basis of the use of antidepressants

When analyzing the data on the subjects' use of medications, it was revealed that all the patients in the TAU group (*n* = 12) were on antidepressive medication, but in the DMT group there were nine patients, who were not taking antidepressants, leaving 12 with antidepressants. Table 5 presents the differences that can be observed when the subjects are grouped on the basis of the DMT intervention and the use of medication.

**Table 5:** **Differences between outcomes in the DMT and TAU groups when the subgroup distribution is based on DMT intervention and taking antidepressants**.

**Variable**		**DMT group, pts taking antidepressants *n* = 12**		**DMT group, no antidepressants *n* = 9**		**TAU group, pts taking antidepressants *n* = 12**	
Mean duration of the treatment period		17 months		10 months		15 months	
Years since first episode of depression (mean)		10 years		5 years		6 years	
**INVENTORY**		*M*	*SD*	*M*	*SD*	*M*	*SD*
BDI-II	Pre	25.58	10.43	24.22	13.16	32.50	7.60
	Post	18.02	13.76	11.55	11.92	28.97	8.65
	Follow-up	19.59	11.88	13.36	14.77	29.66	9.85
HADS	Pre	20.67	7.00	21.00	9.13	24.58	4.65
	Post	15.67	10.57	11.00	9.06	23.54	6.47
	Follow-up	16.96	10.01	11.22	8.57	23.15	7.75
SCL-90	Pre	1.45	0.67	1.30	0.86	1.59	0.41
	Post	1.06	0.80	0.81	0.63	1.58	0.37
	Follow-up	1.03	0.75	0.77	0.54	1.53	0.55
CORE	Pre	17.54	5.53	16.13	7.95	20.65	3.35
	Post	13.44	7.79	10.21	7.70	20.05	4.55
	Follow-up	13.36	7.07	10.77	6.50	19.78	6.04

The duration of the participants' illness, the length of the current treatment period, and the measurements score level differed according to the use of antidepressant medication. Compared to no-antidepressants patients, patients taking antidepressive medications had suffered longer from their illness and had more severe psychiatric symptoms at the pre-measurement point. The TAU group participants on antidepressive medication had the most severe psychiatric symptoms in this material. However, the mean duration of their illness and the length of the current treatment period were shorter than in the subgroup of DMT antidepressant users. Since medication could have affected the results we decided to conduct additional analyses. We were especially interested to ascertain, if the DMT group on medication showed a different change pattern from that in the TAU group (on medication). Further, we were also interested in comparing the members of the DMT group with medication and without medication.

Wald test showed that the DMT group with medication changed differently from the TAU group (on medication) during the intervention regarding the scores on SCL-90, Wald test = 13.46, *df* = 2, *p* = 0.001. In this comparison, the change was statistically significantly different during the intervention period (Estimate = −0.378, *p* = 0.008), but not during the follow-up period.

The HADS scores showed a tendency for a statistically significantly different change pattern when comparing the DMT with no medication and the TAU group (Wald test = 5.472, *df* = 2, *p* = 0.06). In this comparison, the change was statistically significantly different during the intervention period (the Estimate = −8.936, *p* = 0.026). During the follow-up period there was no statistically significant change.

In all other comparisons Wald test did not reveal any statistically significant difference. As there were no statistically significant differences between the score changes of the DMT group with no medication and DMT with medication subgroups, DMT appears to be effective whether the patient is taking antidepressive medication or not.

At the post-measurement, assessing the clinical significance of the changes after the intervention period, the greatest improvements in the condition appeared in the group of DMT participants who were not on antidepressant medication (see Supplementary Table 2). In this group, the within-group pre to post effect sizes ranged from *d* = 0.56 to *d* = 1.07, i.e., from small to medium. The effect sizes in the pre- follow–up measurements comparison ranged from small to large, *d* = 0.62–1.10. The DMT participants on antidepressants had also clearly improved, but the within-ES changes were slightly smaller than for the DMT participants not on antidepressants. In the DMT group on antidepressants the range of effect sizes (*d*) was 0.59–0.76 at the pre-post measurements comparison, and at the pre-follow-up comparison the range was from *d* = 0.53 to *d* = 0.71; thus in this group the ESs were small. In the TAU group, where all the patients were on antidepressant medication, the changes in the scores during the data collection time were minor. The range of within-group effect sizes (*d*) was 0.02–0.47.

## Discussion

This study investigated the effect of adding dance/movement group therapy (DMT) to the treatment of psychiatric outpatients with a diagnosis of depression. Compared to the TAU, adding DMT seemed to improve the effect of the treatment. There was a tendency for the effect of DMT to be slightly better with patients who were not taking antidepressive medication.

Between-group effect sizes between the DMT + TAU and TAU indicated medium or large differences (*d* = 0.60–0.85) in the four measures used in this study in favor of the DMT + TAU. In addition, the within-group effect sizes were considerably larger among patients attending to the DMT group. This suggests, that the favorable changes observed when the DMT was added to the TAU may have clinical significance. However, more studies are needed to confirm the clinical effects of DMT.

The indication of a statistically significantly greater improvement between the DMT + TAU and TAU groups appeared in the SCL-90 measuring psychiatric symptoms and HADS measuring depression and anxiety symptoms. In these self-evaluation assessments, the verbal content of the statements is geared toward bodily felt sensations, symptoms, and emotions. In the SCL-90 one third of the questions refer to somatization or phenomena that relate to autonomous nervous system arousal. This may be one reason why the change was expressed more clearly through these measurements. In addition to these changes, the DMT group showed favorable changes, although not statistically significant, in symptoms of depression (BDI-II) and global distress (CORE-OM). These observations are in line with the study by Punkanen et al. (2014) using a similar DMT group intervention. In their study the mean decrease on the BDI from baseline to post-measurement was 11.17 points compared to 10.11 points in the present study. Both these studies produced a similar favorable outcome in the treatment of depression. Punkanen et al. (2014) used a 20-session group intervention provided twice a week while the present study applied a 12-session intervention. This suggests that favorable changes could also be achieved using a shorter DMT group intervention.

The observations made in this study are also in accordance with the previous reviews by Meekums et al. (2015), Koch et al. (2014), and Papadopoulos and Röhricht (2014). These suggested positive effects of DMT on quality of life and on depression and anxiety. One focus in DMT is engaging with movement activity in the here and now. Further, the aim of activity is to be attentive to the movement experiences and to develop the skills to be aware of experiences, and to communicate about them in words (Meekums, 2002; Koch and Fischman, 2011; Nolan, 2014). Thus DMT involves experiential exercises including mindfulness skills and attention training. There are several other studies suggesting that this type of training, which includes experiential exercises, could be beneficial to the patients (Hayes et al., 2011; Michalak et al., 2012; Horst et al., 2013; Payne, 2015). It could also be speculated that DMT increases psychological flexibility, which has been shown to be associated with wellbeing and quality of life (Hayes et al., 2011; Keng et al., 2011), as the skills for observation, reflection and body state modulation improve. Thus, given that DMT is a useful intervention method for patients with depression symptoms, more studies are needed to examine the possible mechanism of change.

A tendency was observed for the greatest improvement the be achieved when the patient participated in the DMT group and was not on antidepressive medication. However, it should be noted that the patients in the DMT group without or with antidepressant medication benefited from the intervention, and no statistically significant differences were observed between the groups. Thus, more studies are needed to investigate the impact of DMT interventions with or without medication. The importance of observing medication in the treatment is emphasized by the fact that the more difficult symptomology appears to go along with more complex diagnosis set, longer treatment period, and taking of medication. We observed that those patients not taking medication had typically had current treatment periods under 6 months (67% of the patients) and only one diagnosis (44% of the patients). Those patients, who used medication at the pre-measurement, had typically two or more diagnosis (63% of the patients) and had more than 6 months of treatment (63% of patients).

When comparing DMT + TAU to TAU among patients on antidepressant medication, it was observed that all the four outcome measures tended to improve more in the DMT group, with especially SCL-90 showing significantly larger change. It is of particular interest that at the pre-measurement point in the DMT group, the patients on antidepressive medication and those without antidepressive medication had a fairly similar level of symptoms, but the score differences between these two subgroups had clearly increased at the post-measurement, in favor of no antidepressants sub-group. The question arises as to whether the DMT participants on antidepressants had a more difficult type of depression and the medication had alleviated their symptoms so that their symptom scores were on the level of a less complicated depression at the pre-measurement point. If this was the case, it could be assumed that the smaller score changes after the intervention could have been due to the more difficult type of depression.

This study has limitations to be born in mind when drawing conclusions from the results. One concern is the use of self-evaluation measures only, and the lack of movement based assessment of the effects of the intervention. Videotaping the sessions was not part of the usual clinical practice at this clinic, and the goal was to study the natural clinical practice. Without video recordings it is difficult to produce any reliable movement assessment of the four groups. Even with video recordings, movement observation of group activity would have been challenging to carry out reliably.

The participants joined the research groups on the basis of self-selection. They were not randomly divided among the groups. Thus, we cannot ignore the possibility that the selection bias has affected the results. In fact, at the pre-measurement point the TAU group reported significantly higher value for depression symptoms and global distress compared to the DMT + TAU. On the other hand, the DMT group had a slightly longer history of illness, more frequently two diagnoses and more frequently an experience of psychotherapy than the TAU group patients. Also, as more patients in the DMT group had experience of psychotherapy, it is possible that DMT attracts patients who are positively disposed to therapeutic work, willing and able to use self-reflection and interaction as means for their recovery. Both the DMT and the TAU group participants may have had expectations about the treatment they received. As we did not systematically assess their expectations, we can draw no conclusions of the impact of expectations on the results.

Further, the follow-up time was relatively short (3 months), thus in light of the current data it is difficult to draw firm conclusions about the long-term effects of DMT. Another limitation is the small number of participants included in the study. In the TAU group there was a fairly high drop-out rate. However, we applied hierarchical linear modeling in data analyses, since it included all the patients who started the treatment. According to the patient records, all the patients who left the research did continue their treatment at the psychiatric clinic over the study period. No data were collected about their reasons for leaving the study.

The TAU patients were not interested in joining the DMT group, but this study offers no information about their reasons for this. Compared to the TAU group, a higher percentage of the DMT group patients continued in the study. This prompts a question, whether the participation in the DMT group, personal commitment and joining the interaction supported the motivation for treatment and also the alleviation of depression. If this was the case, DMT seems to offer a suitable social context to be utilized in health care to offer new interactional experiences and learning through them.

The TAU did not significantly improve the patients' wellbeing. This study suggests that experiential treatment methods such as DMT could improve the effects of treatment. However, not all clients want to join a DMT group as was observed in this study. In the future, more attention could be devoted for increasing patients' motivation for experiential and action based treatment methods.

The results indicated that adding DMT to TAU is beneficial in the treatment of patients with depression. These results encourage the use of creative, interactive, psycho-physical, and experiential therapy interventions in the treatment of depression.

### Conflict of interest statement

The authors declare that the research was conducted in the absence of any commercial or financial relationships that could be construed as a potential conflict of interest.

## Footnotes

^1^http://www.who.int/healthinfo/statistics/GlobalDALYmethods.pdf

^2^http://www.findikaattori.fi/fi/76

^3^Käypähoitosuositus, http://www.kaypahoito.fi/web/english/guidelineabstracts/guideline?id=ccs00062&suositusid=hoi50023

^4^e.g., http://www.ukkinstituutti.fi/filebank/64-physical_activity_pie.pdf; www.health.gov/paguidelines/guidelines/default.aspx#toc
