# Supplementary material for: A dance movement therapy group for depressed adult patients in a psychiatric outpatient clinic: effects of the treatment
Source: Front Psychol. 2015 Jul 10;6:980. doi: 10.3389/fpsyg.2015.00980 (PMC4498018; doi:10.3389/fpsyg.2015.00980)
Supplement: Supplementary file 1 [file Table1.DOCX]

**Supplementary Material**

**A dance movement therapy group for depressed adult patients in psychiatric outpatient clinic: Effects of the treatment**

Supplementary Table 1: **Effect sizes (d) for depression (BDI-II), anxiety and depression (HADS), physical and psychological symptoms (SCL-90), and global distress (CORE) at pre-post, and 3-month follow-up (fup): within-group effect sizes (d) and effect sizes (d) between DMT and TAU groups.**

| **Out-come** | **with-in  DMT** | **between DMT and TAU** | **with-in TAU** |
| --- | --- | --- | --- |
| **BDI-II** |  |  |  |
| pre-post | -0.87 | -0.67 | -0.47 |
| pre-fup | -0.75 | -0.60 | -0.37 |
| **HADS** |  |  |  |
| pre-post | -0.92 | -0.97 | -0.23 |
| pre-fup | -0.83 | -0.79 | -0.31 |
| **SCL-90** |  |  |  |
| pre-post | -0.57 | -0.70 | -0.02 |
| pre-fup | -0.62 | -0.67^a^ | -0.15 |
| **CORE** |  |  |  |
| pre-post | -0.76 | -0.85 | -0.18 |
| pre-fup | -0.71 | -0.73 | -0.26 |
