# Supplementary material for: A dance movement therapy group for depressed adult patients in a psychiatric outpatient clinic: effects of the treatment
Source: Front Psychol. 2015 Jul 10;6:980. doi: 10.3389/fpsyg.2015.00980 (PMC4498018; doi:10.3389/fpsyg.2015.00980)
Supplement: Supplementary file 2 [file Table2.DOCX]

Supplementary Material

**A dance movement therapy group for depressed adult patients in psychiatric outpatient clinic: Effects of the treatment**

Supplementary Table 2: **A comparison of antidepressants use: within-group effect-sizes (d) and effect sizes between DMT and TAU groups for depression (BDI-II), anxiety and depression (HADS), physical and psychological symptoms (SCL-90), and global distress (CORE) at pre-post, and 3-month follow-up (fup)**

| **Out-come** | **with-in  DMT** | **between DMT and TAU** | **with-in TAU** |
| --- | --- | --- | --- |
| **BDI-II**  **With antidepr.** |  |  |  |
| pre-post | -0.73 | -0.44 | -0.47 |
| pre-fup  **No antidepr.**  pre-post  pre-fup | -0.58  -0.96  -0.83 | -0.35  -0.85  -0.79 | -0.37 |
| **HADS**  **With antidepr.** |  |  |  |
| pre-post | -0.71 | -0.67 | -0.23 |
| pre-fup  **No antidepr.**  pre-post  pre-fup | -0.53  -1.07  -1.10 | -0.38  -1.24  -1.21 | -0.31 |
| **SCL-90**  **With antidepr.** |  |  |  |
| pre-post | -0.59 | -0.69 | -0.02 |
| pre-fup  **No antidepr.**  pre-post  pre-fup | -0.63  -0.56  -0.62 | -0.65^a^  -0.71  -0.69 | -0.15 |
| **CORE**  **With antidepr.** |  |  |  |
| pre-post | -0.74 | -0.77 | -0.18 |
| pre-fup  **No antidepr.**  pre-post  pre-fup | -0.71  -0.76  -0.68 | -0.67  -0.89  -0. 85 | -0.26 |
| ^a) Wald-test = 13.46,  p = 0.001^ |  |  |  |
