# Supplementary material for: A dance movement therapy group for depressed adult patients in a psychiatric outpatient clinic: effects of the treatment
Source: Front Psychol. 2015 Jul 10;6:980. doi: 10.3389/fpsyg.2015.00980 (PMC4498018; doi:10.3389/fpsyg.2015.00980)

A dance movement therapy group for depressed adult patients in psychiatric outpatient clinic: Effects of the treatment

1. Supplementary Figure

Figure A: Flow chart of the research material collection at the psychiatric unit 2011-2013

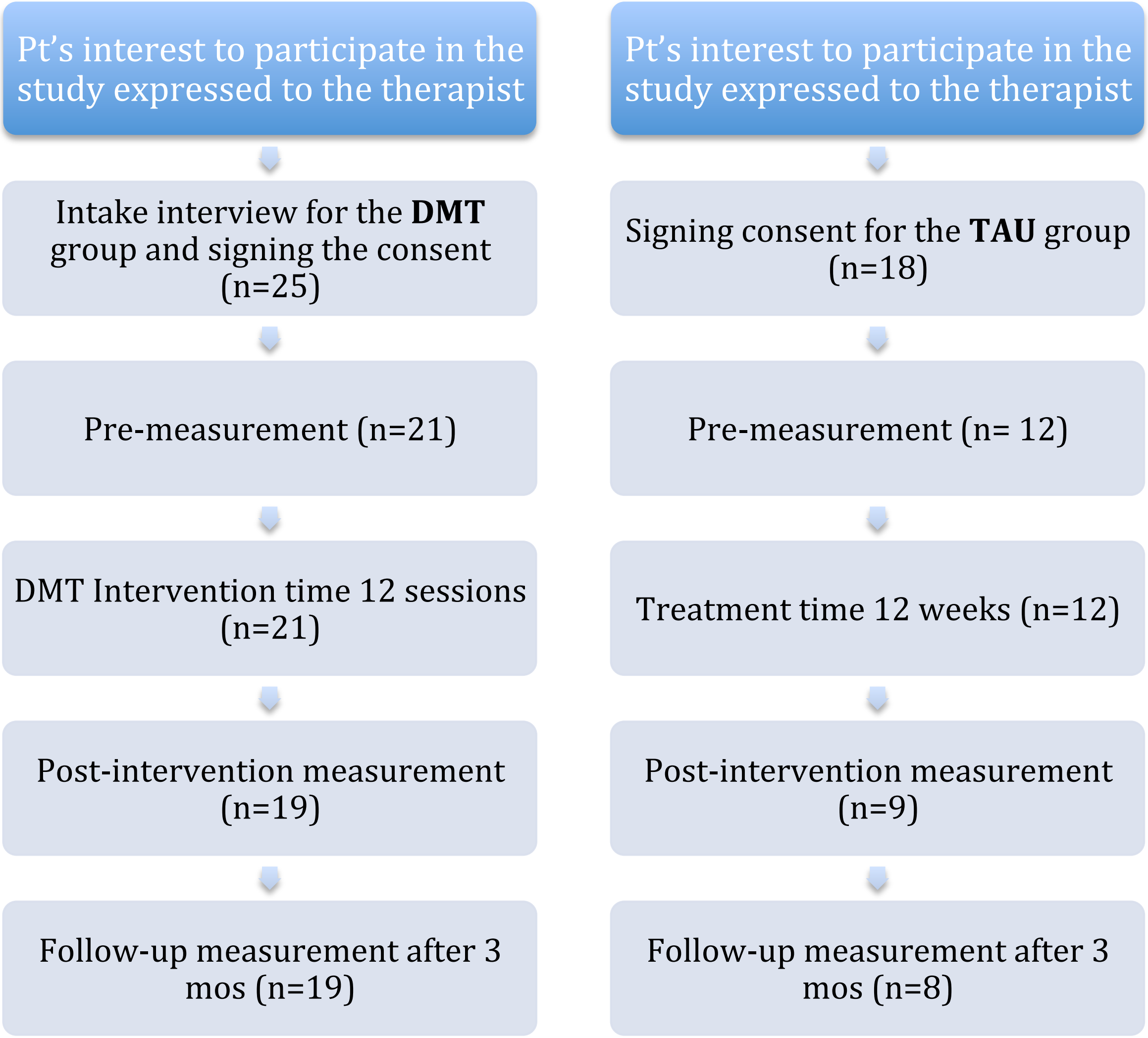

Supplement: Supplementary file 3 [file Presentation1.PDF]
